# Emotional Connectedness to Nature Is Meaningfully Related to Modernization. Evidence From the Meru of Kenya

**DOI:** 10.3389/fpsyg.2018.01789

**Published:** 2018-09-26

**Authors:** Michalina Marczak, Piotr Sorokowski

**Affiliations:** Institute of Psychology, University of Wrocław, Wrocław, Poland

**Keywords:** emotional connectedness to nature, modernization, non-western societies, indigenous environmentalism, human relationship with nature, Biophilia hypothesis

## Abstract

The aim of this study was to investigate an affective relationship with the natural environment in a non-western society and to determine its links with modernization. Emotional connectedness to nature, a significant predictor of nature-protective behavior, was assessed in a sample of 99 members of the Meru people of Kenya, recruited in places supposedly varying regarding their level of modernization: small market towns, farming villages, and a remote pastoralist settlement in the bush. The participants answered questions concerning their level of emotional affinity toward the natural environment and their lifestyle. The results show that feelings toward the natural environment in the studied population were, in general, positive. Such findings support the universality of the Biophilia hypothesis and are promising in the light of extant literature on the links between connectedness to nature and concern for the natural environment. Surprisingly we also found that a more traditional lifestyle was negatively related to emotional connectedness to nature. These findings suggest that contact with nature under conditions of direct dependence on the natural environment may have a different influence on people’s feelings toward nature than in the west. Contrary to the common view, we conclude that the impact of modernization on non-western people’s affective relationship with nature might have been unduly demonized.

## Introduction

Since the industrial revolution, human pressure on the natural world has been growing, leading to global environmental degradation. It was suggested that the ecological crisis is inherently connected with western science, industrial capitalism and culture based on the biblical teachings that humans are to dominate nature (Gottlieb, 1996). Following this line of thought, it is treated as a sort of common knowledge that across the world non-western non-industrialized people live in greater harmony with the natural environment than people in industrialized societies (e.g., Gadgil et al., 1993). From dozens of publications on the subject, we took a closer look at some examples.

For instance, the people of Garhwal Himalaya in India have conserved their sacred sites for many centuries, paying a great deal of attention to the protection of biodiversity in the region (Anthwal et al., 2010). Integrated knowledge about their environments and beliefs that see nature as powerful and sacred are believed to have enabled various groups of Canadian First Nations to live in balance with their local environments for thousands of years (Turner et al., 2000), whereas the Tibetan Baima’s strong adherence to their traditional beliefs was recognized to play a key-role in biodiversity conservation and management of natural resources in their region (Luo et al., 2009).

There exists extensive evidence, however, suggesting that traditional societies might not necessarily be engaged in nature protection (e.g., Hames, 2007). The insignificant negative impact that some non-industrialized societies have had on their natural environments may stem from their small populations, lack of technology which would enable mass-degradation of nature and the absence of external markets which would put further pressure on natural resources (LeBlanc and Register, 2004).

Whether non-industrialized societies deliberately care for balance with the natural environment or not, the topic of the close relationship with nature among these people remains prevalent in western discourses (Berkes, 2018). Such a special bond with nature is in line with the much discussed Biophilia hypothesis which states that humans have an innate need and propensity to affiliate with the natural world, and for this reason they are prone to express pro-environmental concern (Kellert and Wilson, 1995). Indeed, an affective bond with nature was shown to be a significant predictor of nature-protective behavior (e.g., Hinds and Sparks, 2008; Geng et al., 2015).

Evidence from various industrialized societies suggests that, overall, people have positive feelings toward the natural world (see **Table 1**). To the best of our knowledge, no studies have yet examined in a quantitative way people’s emotional relationship with nature outside the WEIRD populations (western, educated, industrialized, rich, and democratic, in the terminology of Henrich et al., 2010). Such an investigation could test the universality of the Biophilia hypothesis. Moreover, if the studied society is, to varying degrees, affected by modernization, its examination directly answers the question of the impact of western culture on people’s feelings toward the natural environment. Additionally, it can shed light on the potential willingness of people to engage in nature protection.

**Table 1:** Mean connectedness to nature in selected populations measured with the Connectedness to Nature Scale (Mayer and Frantz, 2004) on a five point Likert scale.

Sample and country (study)	*n*	*M*	*SD*
Inhabitants of Vienna and its vicinity, Austria (Cervinka et al., 2012)	547	3.55	0.7
High school students in Tuscany, Italy (Di Fabio and Bucci, 2016)	144	4.57	0.79
Farmers, Australia (Gosling and Williams, 2010)	141	3.9	0.66
Secondary school students, Singapore (Leong et al., 2014)	138	3.61	0.52
Portuguese adults in the metropolitan area of Lisbon, Portugal (Loureiro and Veloso, 2014)	282	3.42	0.66
Students and general inhabitants of Madrid, Spain (Olivos et al., 2011)	247	3.43	0.44
Park visitors in Bogota, Colombia (Scopelliti et al., 2016)	300	3.69	0.5
Adults, Great Britain (Swami et al., 2016)	380	3.9	0.59
Adults, United States (Zhang et al., 2014)	1108	3.59	0.83
University students, United States (Zhang et al., 2014)	151	4.54	0.88

For these reasons, we conducted a study in a sample of the Meru, an indigenous East African population who vary in terms of modernization. In accordance with the Biophilia hypothesis, we predicted that people in the studied population will overall have positive feelings toward nature. Our second hypothesis was that modernization will be associated with a decrease in emotional connectedness to nature.

## Materials and Methods

### Participants

The present study was conducted among the Meru people, the indigenous inhabitants of central Kenya. We analyzed data obtained from 99 participants: 49 women aged between 16 and 92 years (*M* = 40.82, *SD* = 20.6) and 50 men aged between 15 and 85 (*M* = 37.62, *SD* = 17.47). The Meru are mostly agriculturalists practicing terrace agriculture. Although there exist numerous places where people follow traditional lifestyle either as small-scale farmers or semi-nomadic herders much like their ancestors, the market towns of the Meru County are influenced to a large extent by western economy and culture (Thomas, 1995).

The selection of the sample was intentional and aimed to reflect the differences in the level of modernization of the subjects. We surveyed inhabitants of a semi-nomadic remote settlement in the bush (*n* = 31), residents of villages up to 60 min ride away from the towns (*n* = 34), and town-dwellers (*n* = 34). People in the settlement in the bush were pastoralists with little access to modern media or the market economy. People in the second group were mostly farmers who partially participated in the market economy and had some access to modern media (especially to cell phones and radio). The town-dwellers functioned entirely in the market economy, and had access to modern media, basic healthcare and schooling. It is worth noting, however, that half of the participants attended only a few years of school and many received no formal schooling at all.

### Procedure

The potential participants were invited to take part in a research project about people’s feelings toward the natural environment. They were told that the researchers would ask 14 questions about the way the respondent feels about nature and a few more questions about their age and habits. All participants provided informed consent before study inclusion and were instructed they could quit the procedure at any time. The consent was oral due to high levels of illiteracy in the region. The study complied with the Declaration of Helsinki. We also obtained the approval of the Institutional Ethics Committee of the relevant university. Furthermore, the study protocol and consent procedure received approval from the head of the local Meru community.

The study was conducted in the Meru language in cooperation with an interpreter from the Meru community. The participants were recruited through opportunistic sampling in the areas mentioned above. Approximately 10% refused to participate.

We assessed participants’ level of modernization based on questions about their place of residence. Additionally, we asked about frequency of visits to the market towns in the past 3 months, years of schooling and TV exposure per week which are common measures of modernization among aboriginal populations (Godoy et al., 2005; Cheung and Kwan, 2009; Sorokowski et al., 2014).

To assess emotional connectedness to nature, we used a questionnaire based on the Connectedness to Nature Scale (CNS, Mayer and Frantz, 2004). The original scale is a popular tool to assess subjects’ perception of feeling emotionally connected to the natural world and is considered to be a good predictor of pro-environmental behavior (Mayer and Frantz, 2004). Fourteen original questions from CNS were discussed with four representatives of the Meru, two women and two men, and developed so that they were comprehensible to an average member of the community. We made every effort to make the questions sound intelligible and we illustrated them with particular instances (see the **Supplementary Material**). Next, the questions were translated into Meru (using the back-translation procedure). The subjects answered the questions on a five-point Likert scale ranging from 1 (strongly disagree) to 5 (strongly agree).

## Results

The following statistics were performed in R Studio Version 3.2.4. The value of Cronbach’s coefficient alpha of the Meru connectedness to nature scale was initially 0.58, therefore we decided to remove the items that presented the weakest correlations. After removing four items (items: 4, 6, 11, 14, see the **Supplementary Material**), the value of Cronbach’s coefficient alpha was rendered acceptable for a scale of this length (0.69). In order to check the construct validity of the connectedness to nature scale adapted for the Meru sample, we performed Confirmatory Factor Analysis (CFA). The model was confirmed a single factor model. We obtained good fit indices (χ^2^ (29) = 36.65, RMSEA = 0.05, SRMR = 0.11, CFI = 0.95). The mean score for each studied variable, as well as correlations between the corrected Meru connectedness to nature scores, and the indicators of modernization, along with the intercorrelations between indicators of modernization, are shown in **Table 2**.

**Table 2:** Correlations between connectedness to nature score and indicators of modernization, and mean scores for each variable.

	1	2	3	4
1. Connectedness to nature	–			
2. Visits in the town	0.36^∗∗∗^	–		
3. TV exposure	0.28^∗∗^	0.54^∗∗∗^		
4. Years of schooling	0.19^∗^	0.46^∗∗∗^	0.52^∗∗∗^	–
5. Place of residence	0.38^∗∗∗^	0.6^∗∗∗^	0.52^∗∗∗^	0.51^∗∗∗^
Mean (SD)	4.05 (0.53)	47.17 (40.42)	3.09 (3.29)	7.54 (5.12)

*Signif. codes: ^∗∗∗^p < 0.001; ^∗∗^p < 0.01; and ^∗^p < 0.05.*

To test the hypothesis that people in the studied population have, in general, positive feelings toward nature, we performed a Wilcoxon rank sum test between the mean level of connectedness to nature in our sample and the baseline level of three which indicates moderate endorsement of nature. There was a statistically significant difference between the mean score in our sample and the baseline level (*W* = 3800, *p* < 0.001).

To approximate whether our sample differs from the western populations reviewed in the introduction, we ran a Wilcoxon rank sum test between the mean level of connectedness to nature in our sample and the averaged connectedness to nature score from the reviewed studies. This time, we observed no significant difference between the means (*W* = 660, *p* = 0.08).

**FIGURE 1:**
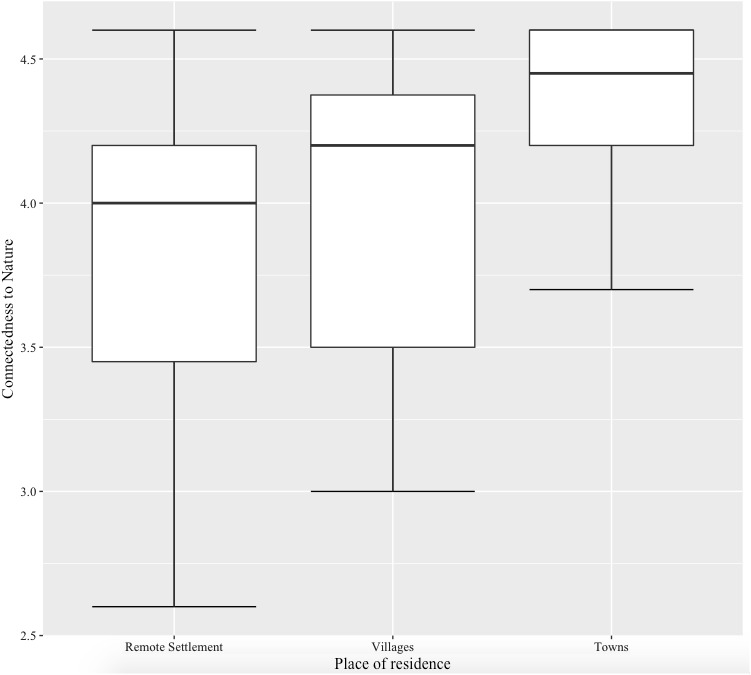
Graphical representation of analysis of variance (ANOVA) with participants’ place of residence (independent variable) and emotional connectedness to nature score (dependent variable).

To verify whether the place of residence of the participants was associated with their emotional connectedness to nature we conducted one-way ANOVA with participants’ place of residence as the grouping variable and their emotional connectedness to nature score as the dependent variable. There was a significant effect of place of residence on the level of connectedness to nature, *F*(2,97) = 8.68, *p* < 0.001, ω^2^ = 0.13. Tukey’s *post hoc* tests revealed significant differences in connectedness to nature between towns and both the remote settlement and villages (towns-remote settlement: *p* < 0.001, *d* = 1.06 towns-villages: *p* = 0.015, *d* = 0.77). These differences are presented graphically in **Figure 1**.

Multiple regression analysis with additional indicators of modernization (frequency of visits to the market towns in the past 3 months, years of schooling and TV exposure per week) was used to test if the indicators of modernization predicted participants’ connectedness to nature. Only visits to the market towns had significant effect on connectedness to nature (*B* = 0.003, *SE* B = 0.002, β = 0.3*, p* < 0.05; *F*(3,95) = 5.31, *p* < 0.001, *R*^2^ = 0.14, adjusted *R*^2^ = 0.12, *SE* = 0.49). In the final regression model, we included the above indicators of modernization as independent variables and sex and age as covariates. This model shown that both visits to the market towns and sex were significant predictors of connectedness to nature [*F*(5,93) = 5.26, *p* < 0.001, *R*^2^ = 0.22, adjusted *R*^2^ = 0.18, *SE* = 0.48]. The results of further examination of the individual predictors are presented in **Table 3**. Including place of residence in the model made other variables become statistically non-significant, which empirically supports the conclusion that place of residence encapsulates the relevant aspects of modernization at stake in connectedness to nature.

**Table 3:** Results of the multiple regression with indicators of modernization, sex and age as predictors and connectedness to nature as the dependent variable.

	*B*	*SE B*	β	*p*
Constant	3.87	0.22		<0.001
Visits to the market towns	0.004	0.001	0.27	<0.05
Years of schooling	0.01	0.01	0.11	0.38
TV exposure	0.03	0.02	0.2	0.1
Sex	−0.24	0.1	−0.23	<0.05
Age	0.01	0.003	0.19	0.07

## Discussion

The current study sought to examine the emotional bond with nature in a non-western society and its links with the influence of modernization in a sample of the Meru, an indigenous East African population who vary regarding their lifestyle. We hypothesized that the Meru will overall have positive feelings toward nature. Moreover, we predicted that an increase in modernization will be associated with a decrease in their emotional connectedness to nature.

In line with the first hypothesis, the feelings toward the natural environment in the studied population were, in general, positive. Such results are in favor of the Biophilia hypothesis which states that humans have a universal innate affinity with the natural world (Kellert and Wilson, 1995). What is more, when compared to the studies presented in the introduction, the endorsement for nature in the Meru was not much different from the feelings toward nature measured with CNS in industrialized populations. It must be noted, however, that we used a modified version of CNS, thus such a comparison should be treated with caution. At the same time, there exist over a dozen measures of people’s subjective relationship with nature (Capaldi et al., 2014). These measures are often used interchangeably in the extant literature, since they are all highly correlated with each other and with other relevant constructs (i.e., personality, environmental attitudes) in a similar way (Tam, 2013). Therefore, despite using a different scale, to state that the studied population show similar feelings toward nature as other studied populations is justified.

These findings are promising in the context of links between affinity with nature and pro-environmental behavior which were observed in industrialized populations (Schultz, 2001; Mayer and Frantz, 2004; Schultz and Tabanico, 2007; Nisbet et al., 2009; Gosling and Williams, 2010; Geng et al., 2015). It remains obscure, however, whether such links exist in non-western populations. Further research should focus on linking connectedness to nature to non-western people’s pro-environmental behavior.

Contrary to the second hypothesis, we found that indicators of modernization such as place of residence and frequency of visits to the market towns were positively correlated with emotional connectedness to nature. One explanation for our findings might be associated with a different degree of dependence on the natural environment in the studied population. Far from the towns the contact with nature is inevitable as more traditional people live off their land as horticulturists and pastoralists. Interestingly, findings from western societies indicate that spending time in nature is related to increased affinity toward the natural environment (Mayer and Frantz, 2004; Mayer et al., 2008; Collado et al., 2013; Navarro et al., 2017). However, time spent outdoors in these studies was limited to recreational activities among safe and particularly attractive elements of the natural world, and often associated with free time whereas the contact with nature among the Meru inhabiting remote villages and the pastoralist settlement was constant, and related to, among other things, danger posed by wild animals, experiencing heat and water shortages in the dry season and torrential rains during monsoon time. Perhaps the liberation from direct dependence on nature allows people to appreciate its qualities more.

Another point to consider is that individuals who only occasionally spend time outdoors might be biased to perceive and remember nature’s mostly positive sides. Town-dwellers might associate nature with time off, visiting relatives in the countryside and various gratifications stemming from it as well as with neat images from wallpapers, rather than the harsh realm of snakes and scorpions, water and firewood shortages as well as extreme weather conditions which are experienced daily by the herders. A similar connection between the contextual influences in the natural environment (seasonal and meteorological changes) and feelings toward nature was found in an American sample (Duffy and Verges, 2010). In this sense, nature viewed in a more abstract way might foster cognitive errors that turn people’s feelings in favor of the environment. Future studies should focus on the links between environmental conditions and an affective relationship with nature.

Our study has several limitations. First, the concept of connectedness to nature might not be culturally universal. Nature and humans’ place in it outside the Western world might be perceived in different categories than in the West (Van den Born et al., 2001; Sulemana et al., 2016). The assessment of affective bond with the environment might be therefore laden with an artifact associated with an attempt of studying people from a different culture through the lens of notions constructed in the Western culture. More qualitative ethnographic information would allow for better understanding of Meru’s meanings of relationship with nature.

Another point to consider as limitation of the present study, is the reliance on an exogenous institutional review board’s approval. Although relying on the institutional review board of researchers’ home university seems to be customary in research in traditional societies published in peer-reviewed journals (e.g., Purzycki et al., 2016), it contributes to the problem of bias in accountability that plagues the field of social scientific research regarding populations that have little institutional representation in the academy. As much as obtaining the approval from an ethical review board of the Meru would be the most reliable, it was not possible because such body did not exist. However, in order to ensure that the study protocol was suitably anchored in the cultural context of the Meru, we consulted a number of representatives of the Meru about the form and content of the study.

Due to resource constraints, the Connectedness to Nature Scale was not fully validated for use with the Meru. However, in order to ensure maximum validity, the items were first formulated in the most understandable way in cooperation with four Meru representatives, and consequently translated from English using the back-translation procedure. Moreover, CFA results suggest that the scale used can be considered psychometrically valid. Additionally, as noted by Riley et al. (2017), the external validity of scales developed in one population often overlaps when these measures are used in other populations. Nevertheless, our results should be interpreted carefully.

This research examined the links between modernization and emotional affinity toward nature based on a sample of one ethnic origin – the Meru people of Kenya. In order to determine the relationship between modernization and the impact of western culture on people’s feelings toward the natural environment, more research in traditional populations is needed.

## Conclusion

Our findings provide rare quantitative data on the affective relationship with the natural environment in a non-western society. Whereas overall positive feelings toward nature in the studied population are promising regarding people’s potential willingness to engage in pro-environmental behavior, the positive links between modernization and connectedness to nature which we found suggest that, at least in the studied group, the influence of modernization on non-western people’s emotional bond with the environment might have been unduly demonized. We hope that this paper will bring more attention to people’s psychological relationship with the natural environment outside the WEIRD world.

## Data Availability

The raw data supporting the conclusions of this manuscript will be made available by the authors, without undue reservation, to any qualified researcher.

## Author Contributions

MM and PS conceived and designed the study, analyzed the data, and wrote the manuscript. MM collected the data.

## Conflict of Interest Statement

The authors declare that the research was conducted in the absence of any commercial or financial relationships that could be construed as a potential conflict of interest.
