# Supplementary material for: Emotional Connectedness to Nature Is Meaningfully Related to Modernization. Evidence From the Meru of Kenya
Source: Front Psychol. 2018 Sep 26;9:1789. doi: 10.3389/fpsyg.2018.01789 (PMC6168673; doi:10.3389/fpsyg.2018.01789)
Supplement: Supplementary file 1 [file Table_1.DOCX]

Supplementary Material

Emotional Connectedness to Nature Increases With Modernization. Evidence from the Meru of Kenya.

Michalina Marczak^1*^, Piotr Sorokowski^1^

^1^Institute of Psychology, University of Wrocław, Wrocław, Poland

*** Correspondence:**

Michalina Marczak
michalina.marczak@gmail.com

1. The Meru connectedness to nature questionnaire in the Meru language.

Nukurombwa gucokia biuria bibi uria ukwigua niu biagirite. Gutina gicokio kiama kana kia urongo. Ugitumagira ithimi bibi cokia bibi na umaa buru uria ukombaa.

| 1 | 2 | 3 | 4 | 5 |
| --- | --- | --- | --- | --- |
| buruu arii | kaaba arii | kwina inya kuuga | kabaa ii | buru buru ii |

1. Nimbigagua ndiri na urume na into bia nthiguru biria biri amwe nani? Atii into biu nibiretaga ukiri muturirene jwakwa?

2. Ninkuthugania nthiguru ni umwe na nturaa iria kinya nii ndiri umwee wayoo? Wina mucii, atana ba abagu na arii ba nyankwe, mwekuru na twana? Antuu baa bonthee niba bata mono kiri gwee? Uturone bwakuu kuri na ng’ombe, mburi, mbea, ikwaa na miti? Ni wigagua kinya into bii biri bia bata mono kiri uturo bwakuu?

3. Nimenyaga na ngacokia nkathoo ni untu bwa umee bwa nyomoo ingi iria irii mwoyo? Atii nyomoo ona into bingi nibibuirite birina uume, ng’ombe kumenya iria riria ibwirite kuria na nyomoo ingii gwicithaa maithaa.

4. Jaria maingi nimbigagua ntina urumwe na into biria binthirunkite?

5. Nucithuganagiria uri umwee wa uria uturo bwa thiguru ijii bukari? Ja uria  iria ririjagwaa ni nkurungu, nayo nkurungu ikariwa  ni simba nayo simba yakua ikariwa ni nyonii na mbitii, na biria bigatigara bikanoria miunda ikauma iria ringi ria nkurungu iria chitigi. Nuthuganagia iria umwee wa muturire juu jwa nthiguru?

6. Jaria maingi nimbigagua ndiri na urumwe na nyomoo oo amwe na miti na imera.

7. Nimbigagua ati ndiri umwee wa nthiguru ota uria nthiguru iri oo amwe nani. Atii nimbuirite kumenyera nthiguru kenda nayo iimpa biria kwenda.

8. Ninkuerewa uria mathithia jwakwa jakureta ugaruruku nthiguru. Ninkumenya kuthinja ng’ombe ikamatite igatumaa mbage tujau matuku jakeja, kana ndaithia mwituu ntiona mbau kana mbura kairi?

9. Jaria maingi nwigagua uri umwee wa  muthiurunko jwa uria antu na nyomoo ituraga nthigurune. Into bia nthiguru birina gitumi kiri ugwe?

10. Jaria maingi nwigagua atii into bia nthiguru, antu na nyomoo, nibiithagirwa birina untu bumwee mwoyone?  Ja, bionthe kabiaciarirwe, nibibuirite kuria na bigakinyia bikue?

11. Otauria muti jwithagirwa juri gacigo ka mwituu, nwigagua uri gacigo ka into bionthe bia nthiguru?

12. Riria ndirathuganiria nthiguru, nthuganagia nini mukaria wa into bionthe bia nthiguru? Nini mukaria wa into bionthe biria biri nthiguru ijii? Bintethagiria na mobatu jwakwa jaria ndiri najoo? Nyomoo cigantethagia kiri ngugi ciakwa nayo miti igambejaga mbao na mburaa?

13. Jaria maingi nimbigaua ati ndiri gacunci ka into bia nthiguru biria binthirunkite na ati ntiwa gitumi gukuruka iria riria riri nthiguru kana nyoni cia mwituu?

14. Muturire jwakwa ningwa jutikugwatana na mantu ja nthiguru? Ugima bwakwa bwa mwiri na kugwirua gwakwa gutikugwatana na mantu ja nthii, ja mwituu? Ndomba kwithirwaa ngwiritue kinya miti yagitangwa kana nyomoo cioragwa?

2. The Meru connectedness to nature questionnaire in English.

Please answer the following questions about the way you feel about nature. There is no right or wrong answer. Simply state what you feel at the present moment. You can use the following answers:

| 1 | 2 | 3 | 4 | 5 |
| --- | --- | --- | --- | --- |
| definitely no | rather no | hard to say | rather yes | definitely yes |

1. Do you feel a sense of unity with the natural world that surrounds you? That the trees, plants and animals bring peace to your life?

2. Do you feel at the moment that the natural world, the world of plants and animals, is like a village that you belong to? Do you have a family, brother and sisters, wife or children? Are these people important to you? In your life you have cows, goats and trees. Do you feel that they are important in your life?

3. Do you believe that other living things such as animals and other creatures have knowledge and their own wisdom? Like cows know which grass to eat or other creatures know how to hide from the predators.

4. Right now, would you say that you don’t care about nature?

5. Do you think that you are a part of the cycle of life? Like grass is eaten by an antelope, and the antelope is eaten by a lion and the lion is eaten by the vultures and scavengers and what remains decomposes making the soil fertile for the grass to grow again and the remaining antelope to feed on it. Do you think of yourself as a part of it?

6. Right now, do you feel any affection towards the animals and plants and the land of living beings around you?

7. Do you feel that you belong to the land of animals and plants as much as it belongs to you? Do you believe that what you do to nature can affect you personally?

8. Do you understand how your actions affect the natural world? That for example slaughtering a pregnant cow will make you end up without any calves in the days to come or when you burn or cut down the forest you will not get wood or firewood any more?

9. Do you most of the time feel that you are a part of the nature’s cycle? Are the living things around you important to you?

10. Do you feel that animals and people share a common life force? That they have the same fate meaning that they are born, live and grow and will all die one day?

11. Like the tree is part of the forest, do you feel that you are a part of nature?

12. Do you feel that you rule over the animals and plants? Are they there to serve your needs? Are the trees made for using them for building houses or burning fire? Are the animals there to hunt them and eat them?

13. Do you feel that you are part of nature that surrounds you, a part no more important than the grass on the ground or the birds in the trees?

14. Would you say that your personal life is not affected by what happens to nature? That your health and happiness are not affected by the condition of the natural world? Could you be happy if the trees were cut down or the animals were killed?
